# Genetic evidence identifies a causal relationship between EBV infection and multiple myeloma risk

**DOI:** 10.1038/s41598-025-90479-1

**Published:** 2025-02-21

**Authors:** Jian Li, Rong Tan, Bing Yang, Changpu Du, Jie Tian, Zhu Yang, Dongxin Tang

**Affiliations:** 1 The First College of Clinical Medicine, Guizhou University of Traditional Chinese Medicine, No. 71, Baoshan North Road, Yunyan District, Guiyang, 550001 Guizhou China; 2 Department of Pharmaceutics, The First Affiliated Hospital of Guizhou University of Traditional Chinese Medicine, Guiyang, Guizhou China; 3 Student Management Office, The First Affiliated Hospital of Guizhou University of Traditional Chinese Medicine, Guiyang, Guizhou China; 4 Department of Oncology, The First Affiliated Hospital of Guizhou University of Traditional Chinese Medicine, Guiyang, Guizhou China; 5 Guizhou University of Traditional Chinese Medicine, No. 4, Dongqing Road, Huaxi District, Guiyang, 550025 Guizhou China

**Keywords:** Epstein-Barr virus, Multiple myeloma, Causal association, Mendelian randomization, Tumour biomarkers, Tumour immunology, Biomarkers, Cancer epidemiology

## Abstract

Background: Previous observational studies have suggested a potential association between Epstein-Barr virus (EBV) infection and the development of multiple myeloma (MM), but this relationship is not clear. Therefore, we conducted a systematic Mendelian randomization (MR) analysis to investigate the causal relationship between EBV infection and the risk of MM, while exploring the possible mediating role of immune cells in this association. Methods: The study first conducted a two-sample MR analysis using the MM R11 dataset from the FinnGen Consortium to evaluate the causal relationship between five EBV infection-related antibodies (AEB-IgG, EA-D, EBNA-1, VCA-p18, and ZEBRA) and MM, with validation in the MM R10 dataset. A reverse MR analysis was then performed. For significant results, multivariable MR (MVMR) was used to adjust for the effects of confounding risk factors. Next, a two-step MR mediation analysis was applied to investigate the potential mediating role of 731 immune cell types between positive exposure and MM. Multiple sensitivity analyses were conducted to assess the robustness of the findings. Results: A two-sample MR study found that EBNA-1 antibodies (OR = 1.36, 95% CI: 1.06–1.73; *P* = 0.015) were associated with an increased risk of MM, with similar results observed in the FinnGen Consortium R10 replication study. Although the association did not remain statistically significant after false discovery rate (FDR) adjustment (P_fdr = 0.075), further adjustment for relevant confounders using multivariable MR (MVMR) demonstrated that EBNA-1 antibodies (OR = 1.33, 95% CI: 1.01–1.75; *P* = 0.041) were still significantly associated with an increased risk of MM. Reverse MR analysis indicated no causal effect of MM on EBV-related antibodies. A two-sample MR analysis involving 731 immune cell phenotypes identified 27 potential mediating cell types. Ultimately, two-step MR confirmed that HLA-DR on myeloid dendritic cells (HLA-DR⁺ mDC) serves as a mediating factor, with EBNA-1 antibodies downregulating HLA-DR⁺ mDC, thereby increasing MM risk. Multiple sensitivity analyses supported the robustness of these findings. Conclusion: The findings of this study suggest that EBNA-1 antibodies may increase the risk of MM by downregulating HLA-DR⁺ mDC. This indicates that chronic EBV infection may contribute to an elevated risk of MM. We hope these results provide new insights for future research on the prevention and treatment of MM.

**Supplementary Information:**

The online version contains supplementary material available at 10.1038/s41598-025-90479-1.

## Introduction

Multiple Myeloma (MM) is a clonal B-cell malignancy characterized by the accumulation of malignant plasma cells in the bone marrow^1^. MM accounts for 1% of all cancers and 10% of hematologic malignancies, making it the second most frequently diagnosed hematologic malignancy^2^. Globally, over 187,000 new cases of MM are reported annually, with approximately 121,000 deaths attributed to the disease. Furthermore, both its incidence and mortality rates are on the rise^3^. Despite significant advancements in treatment, including proteasome inhibitors, immunomodulatory agents, and monoclonal antibodies, MM remains an incurable disease, with most patients ultimately succumbing to relapse and drug resistance^4,5^.

The immune system plays a pivotal role in the initiation and progression of MM. Specifically, dysregulation of immune cells can facilitate tumor immune evasion and further tumor development^6^. Studies have highlighted that various immune cell phenotypes, such as T cells, natural killer cells, and myeloid-derived suppressor cells, are critical components of the immunological microenvironment in MM^1,7^. Epstein-Barr virus (EBV), a ubiquitous herpesvirus infecting over 90% of the global population^8^, is well-known for its role in various malignancies, including Burkitt lymphoma^9^, nasopharyngeal carcinoma^10^, and Hodgkin lymphoma^11^. Emerging evidence suggests that EBV may also contribute to the pathogenesis of MM^12–16^. Immune suppression caused by tumors^17^ or aging^18,19^ is one of the important factors contributing to the reactivation of latent EBV infection. However, once EBV infection is activated, it can influence the immune system by modulating B cell function, promoting chronic immune activation, and altering the cytokine network, which may further create a microenvironment conducive to the development of MM^15,20^.

However, most existing studies are based on observational data, which presents significant challenges in translating these findings into effective cancer prevention and control strategies. This is primarily due to the susceptibility of traditional observational designs to various biases, such as residual confounding^21^and reverse causation^22^. Despite statistical and methodological efforts to address these issues, biases often persist^23^, making it difficult for observational studies to reliably establish causal relationships between exposures and outcomes^24^.Mendelian randomization (MR), by leveraging genetic variations in the form of single nucleotide polymorphisms (SNPs) as instrumental variables (IVs), effectively addresses confounding and reverse causation inherent in traditional observational studies, thereby providing more robust causal inferences^25^. For example, some researchers have used MR Analysis to explore the causal relationship between circulating inflammatory markers and cancer^26^. Therefore, we conducted a systematic MR analysis to investigate the causal relationship between EBV infection and the risk of MM, while exploring the possible mediating role of immune cells in this association.

## Materials and methods

### Study design

**Fig. 1:**
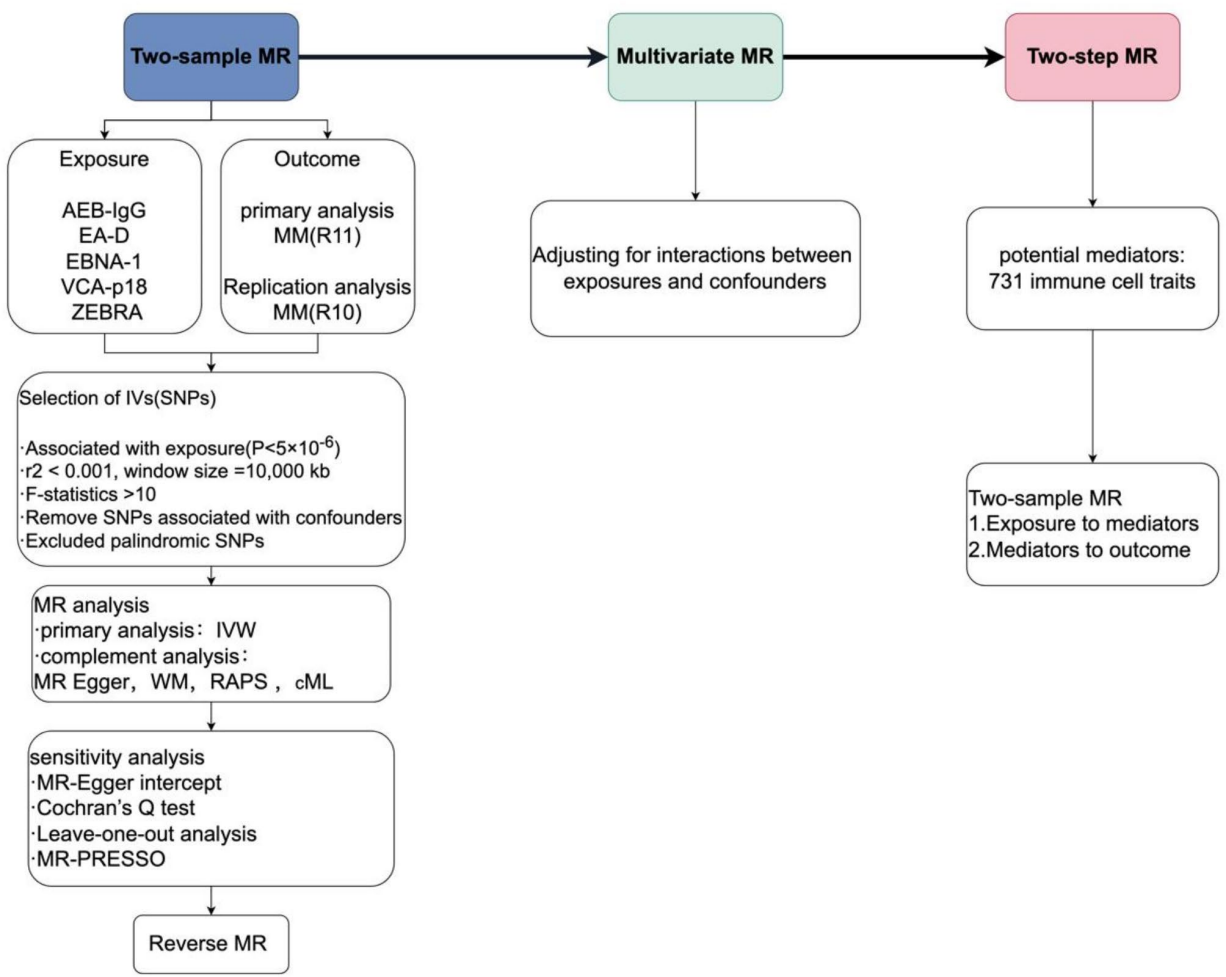
The overview of the study design and flowchart in this study (by Figdraw). SNPs, Single nucleotide polymorphisms; LD, Linkage disequilibrium; MR, Mendelian randomization; IVW, Inverse variance weighted; WM, weighted median; RAPS, Robust adjusted profile score; cML, Constrained maximum likelihood.

MR studies must meet three key core assumptions to ensure its validity^27^ (1) the genetic variant IVs used for MR analysis, must be significantly correlated with the exposure factors studied; (2) the IVs must be independent of potential confounders; and (3) the effect of the IVs on the outcome must be exclusively through the exposure factors, and not by other pathways to directly affect outcomes. Our MR study design met these three core assumptions and followed the STROBE-MR guidelines (Supplementary STROBE-MR-checklist). We first performed a two-sample MR study to assess the causal relationship between 5 EBV-related antibodies (AEB-IgG, EA-D, EBNA-1, VCA-p18, ZEBRA) and MM using the Finnish Consortium’s R11 dataset, validated with R10. Reverse MR analysis followed. For significant results, multivariable MR (MVMR) was applied to adjust for confounding risk factors. A two-step MR explored the potential mediating role of 731 immune cell types between positive exposures and MM. Multiple sensitivity analyses were used to confirm the robustness of the results. The entire study design is shown in Fig. 1.

### Data sources

All data used in the MR analysis were obtained from publicly available genome-wide association studies (GWAS). GWAS data for five EBV infection-associated antibodies (AEB-IgG, EA-D, EBNA-1, VCA-p18, ZEBRA) were obtained from a UK Biobank (UKB) cohort study containing serologic measurements of up to 10,000 cases of infectious diseases and genome-wide genotyping^28^. We obtained MM R11 and R10 versions of the GWAS summary data from the FinnGen Consortium (https://finngen.gitbook.io/documentation/). MM eligible for ICD-O-3 diagnosis with control exclusion for all cancers. The immune cell GWAS data were obtained from an immune cell genetic characterisation study including 3757 Sardinian participants^29^, all data are available from the public GWAS database IEU Open GWAS (https://gwas.mrcieu.ac.uk/, ID: ebi-a-GCST90001391 to ebi-a-GCST90002121). GWAS data for confounding factor obesity were similarly derived from the IUE open GWAS (https://gwas.mrcieu.ac.uk/, ID: finn-b-E4_OBESITY). For more information on the above data, please refer to Supplementary Table 1.

### Instrumental variable selection

We used single nucleotide polymorphisms (SNPs) as IVs for genetic variation. The traditional threshold for selecting genome-wide significant SNPs is *p* < 5 × 10^− 8^. However, this strict criterion can be problematic if an insufficient number of SNPs are identified, potentially reducing the study’s statistical power or, in some cases, leading to exaggerated results^30^. To mitigate this, we adopted a slightly more lenient p-value threshold when necessary, ensuring a minimum of three SNPs met the criteria for IVs to enhance the power to detect significant associations. First, we extracted SNPs with genome-wide significance for exposure in GWAS, for which we chose a threshold of *p* < 5 × 10^− 6^ in the two-sample Mendelian analysis and *p* < 5 × 10^− 8^ in the two-step Mendelian randomization analysis. Second, we excluded SNPs with linkage disequilibrium (LD) (r2 < 0.001, clumping distance = 10,000 kb) to eliminated highly associated SNPs. Third, we harmonize the effect sizes and alleles of SNPs in the exposure and outcome data. To prevent weak instrumental variable bias, SNPs with F-statistics < 10 were removed (F = R2 (n-k-1)/k(1-R2)). To minimise bias due to confounding factors, we screened for SNPs strongly associated with obesity as a confounding factor (*p* < 5 × 10^− 8^) via catalogue sites (https://www.ebi.ac.uk/gwas/). No SNPs associated with obesity were found among the selected SNPs (Supplementary Table 3). Finally, we used SNPs that met all the above criteria as IVs for the MR analysis. The characteristics of the SNPs used in this study are presented in Supplementary Table 2.

### Statistical analysis

We used the inverse-variance weighted (IVW) method as the primary MR analysis. IVW assumes that all genetic instruments are valid and provides a weighted average of the SNP-specific causal estimates. This method offers high statistical power under the assumption that there is no horizontal pleiotropy^31^. Cochrane’s Q-tests were performed to scrutinize SNP-related heterogeneity for each exposure. In the presence of significant heterogeneity (*p* < 0.05), a random-effects IVW (RE-IVW) model was used; conversely, a fixed-effects IVW (FE-IVW) model was used. In addition, we performed a variety of other complementary MR Methods, including MR-Egger, weighted median (WM), Constrained maximum likelihood (cML), and Robust adjusted profile score (RAPS) to bolster the robustness and credibility of the MR outcomes. The MR-Egger method uses the regression intercept as an indicator to test potential multiple effects, and a P value less than 0.05 indicates pleiotropic effects^32^. When more than 50% of the IVs are valid, the results of the WM method are reliable^33^. cML is used to exclude bias caused by correlated and uncorrelated pleiotropy^34^. RAPS allows the inclusion of weak instrumental variables and provides robust statistical estimates for MR through these weak instruments^35^. The leave-one-out method was used in sensitivity analysis to assess the effect of individual SNPs on overall causal estimates. We also applied MR-PRESSO to detect outliers and found that the removal of outliers effectively reduced horizontal pleiotropy. In addition, we performed sensitivity analysis on the MR results using scatter plots and funnel plots. We used the Steiger Test to detected the selected SNPs for potential reverse causality between EBV-related antibodies (AEB-IgG, EA-D, EBNA-1, VCA-p18, ZEBRA) and MM (Supplementary Table 7). To control the false-positive error rate, multiple comparisons in the MR study were adjusted using false discovery rate (FDR) correction. A P_fdr < 0.05 was considered statistically significant, indicating a robust causal relationship between the exposure and the outcome. However, when *P* < 0.05 but P_fdr > 0.05, the result was interpreted as suggesting a potential causal relationship. We performed reverse MR using the same criteria and also performed two-sample MR as a repeat validation using the Finnish R10 version of MM data as the outcome. For causally related exposures and outcomes, we confirmed direct causality by adjusting for the interaction of relevant risk factors and exposures using multivariate MR(MVMR).

To investigate the potential causal mechanisms between EBV infection and MM, we conducted a two-step MR mediation analysis. Previous studies have shown that abnormalities in immune cells are associated with the development of MM^15,20^ and that EBV infection causes immune dysfunction. Thus, we selected 731 immune cell types as potential mediators. First, we applied two-sample MR to examine the causal effect of 731 immune cell types on the MM. Positive results were used as possible potential positive mediators. We then used two-step MR to assess the mediating role of potential positive mediators in the causal relationship between positive exposure and MM.

All statistical analysis and data visualizations were performed with the “TwoSampleMR”, “MRPRESSO”, “Forestplot”, “coloc”, “MRcML” R packages in R software version 4.3.3 (R Foundation for Statistical Computing, Vienna, Austria).

## Results

### Two-sample MR

**Fig. 2:**
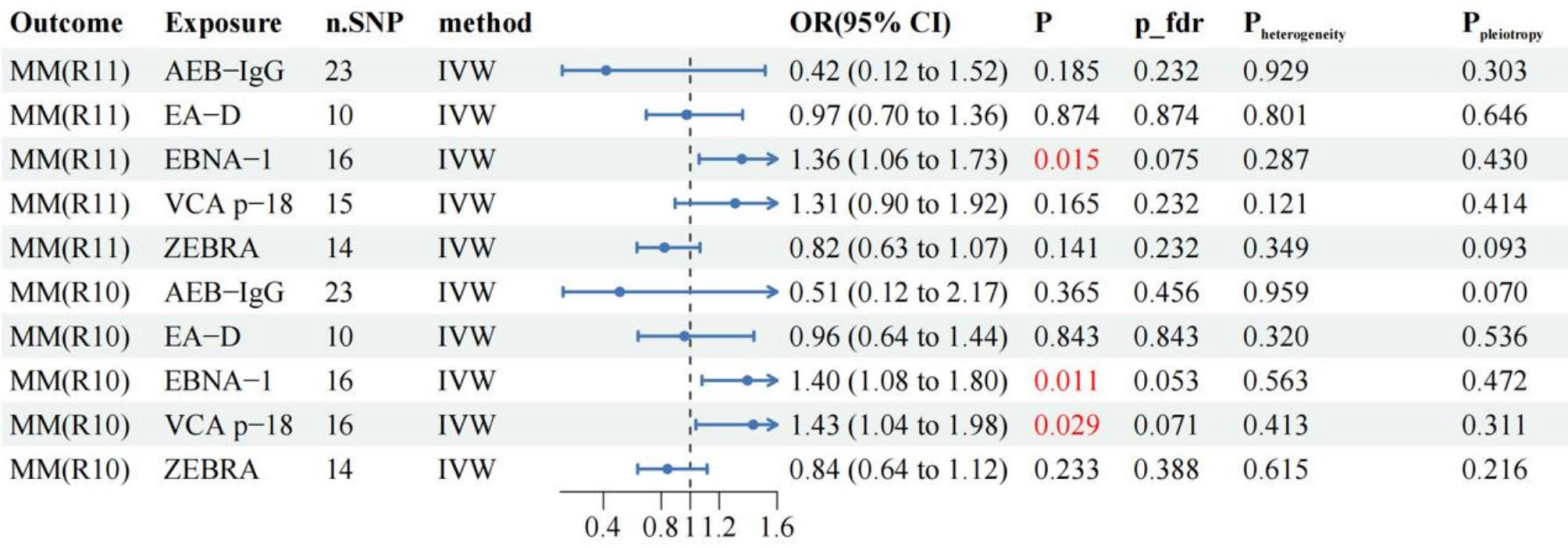
Two-sample MR forest plots for primary analysis and validation analysis.

In the primary analysis of 5 antibodies associated with EBV infection and MM (R11), we screened a total of 78 eligible SNPs for two-sample MR. Details of the selected SNPs are shown in Supplementary Table 2. The results of the two-sample MR analysis showed that EBNA-1 antibody may increase the risk of MM, while other EBV infection-related antibodies were not associated with the risk of MM (Fig. 2). Through Cochran’s Q test (Supplementary Table 6), we did not find heterogeneity in gene IVS related to EBNA-1 (P_heterogeneity_=0.287), so we selected the FE-IVW as the main MR analysis. FE-IVW results showed that EBNA-1 increased the risk of MM (OR = 1.36, 95% CI: 1.06–1.73; *p* = 0.015). WM (OR = 1.49, 95% CI: 1.08–2.04; *p* = 0.015), cML(OR = 1.35, 95% CI: 1.01–1.81; *p* = 0.046) and RAPS (OR = 1.39, 95% CI: 1.03–1.86; *p* = 0.030) methods also yielded results consistent with FE-IVW. However, MR-Egger (OR = 1.62, 95% CI: 0.99–2.64; *p* = 0.075) results showed no statistical significance, but exhibited the same trend. In sensitivity analyses, neither the MR-Egger intercept nor the MR-PRESSO test indicated evidence of pleiotropy (Supplementary Table 5). Additionally, the leave-one-out analysis did not identify any SNPs that introduced significant bias to the results. Funnel plots and forest plots are provided in Supplementary Fig. 1. These sensitivity analyses collectively support the robustness of the findings. Although, the causal relationship between EBNA-1 antibodies and MM risk after FDR correction was not significant (P_fdr = 0.075). However, in replication MR analyses using the Finnish R10 version of MM data, we still found that EBNA-1 antibodies increased the risk of MM (OR = 1.40, 95% CI: 1.08–1.80; *p* = 0.011). And it was also found that VCA-p18 antibody may also increase the risk of MM (OR = 1.43, 95% CI: 1.04–1.98; *p* = 0.029). Sensitivity (Supplementary Table 5) and heterogeneity (Supplementary Table 6) analyses indicated no issues, supporting the robustness of these findings. The replication results align closely with those of the primary analysis using the Finnish R11 version, further confirming the reliability of our results.

We performed reverse MR analysis using SNP selection thresholds consistent with the main analysis. We did not find an inverse causal relationship between MM and antibodies associated with EBV infection. Detailed results are presented in (Supplementary Table 4).

### MVMR

**Fig. 3:**
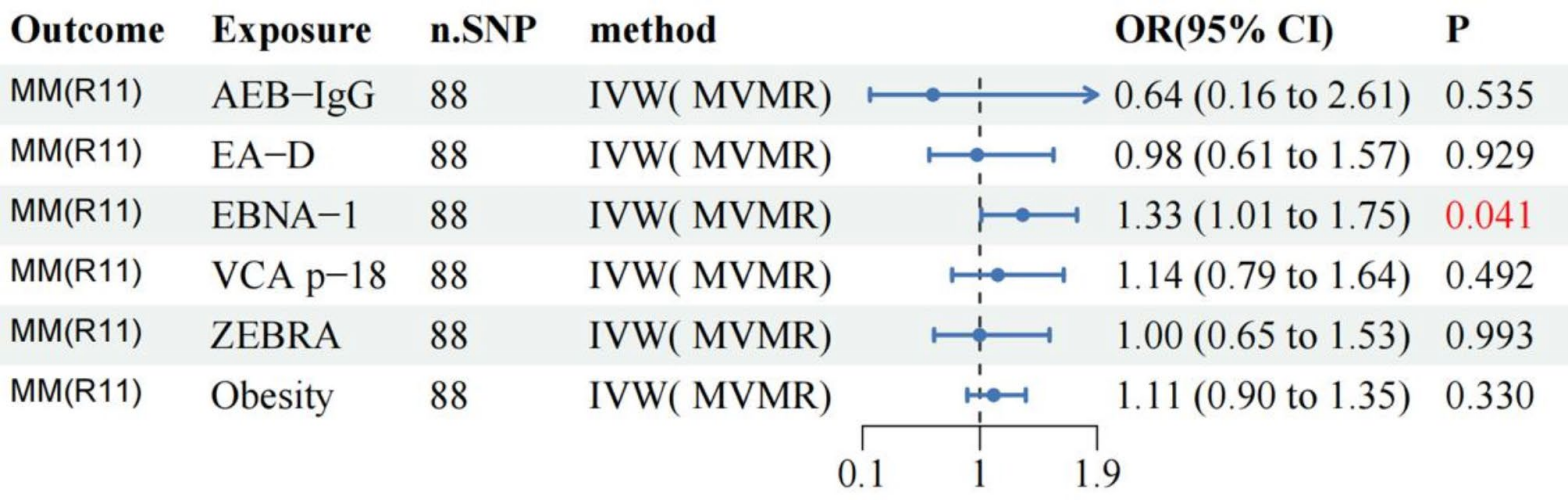
Forest plot of MVMR for EBV infection-associated antibodies and obesity with risk of multiple myeloma (MM).

A previous study found that obesity may be a risk factor for MM^36^, and in order to reduce the impact of this risk factor on MR outcomes, we selected it for MVMR analysis. To mitigate the interactions between EBV-associated antibodies and between confounding factors (obesity), we performed MVMR to explore the direct causal relationship between antibodies and MM. After adjusting for MVMR interactions, the results showed that the effect of EBNA-1 antibody on MM risk was attenuated, but whereas EBNA-1 still increased MM risk (Fig. 3). Other EBV-related antibodies were not found to be associated with MM risk. The MVMR Egger intercept test showed no evidence of horizontal pleiotropy (P_pleiotropy_ =0.522). These MVMR findings further support that EBNA-1 antibody increases the risk of MM.

### Two-step MR

**Fig. 4:**
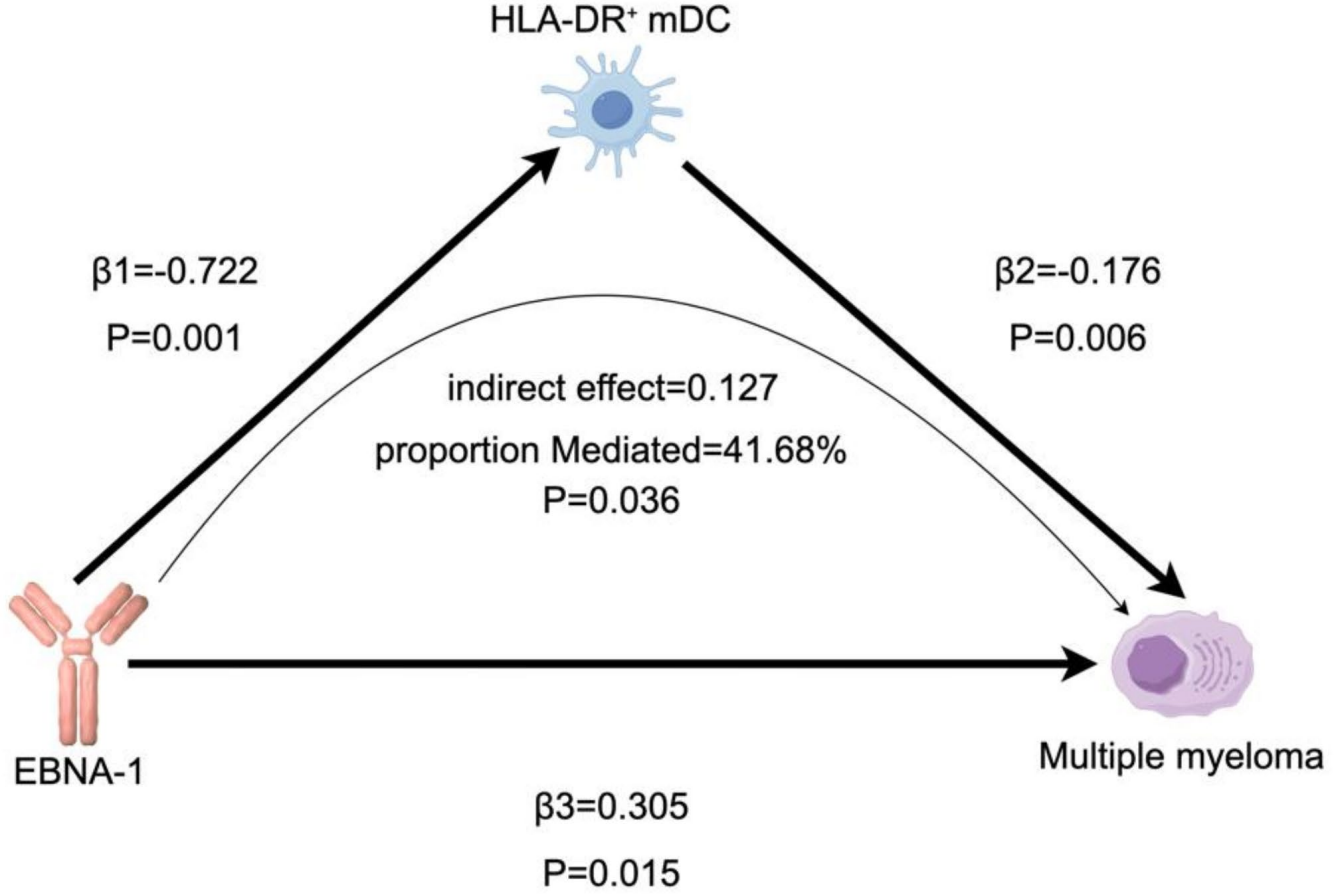
Schematic diagram of mediating effects of HLA-DR on myeloid dendritic cells (HLA-DR⁺ mDC) levels. β1 represents the causal effect of Epstein-Barr virus EBNA-1antibody levels on HLA-DR⁺ mDC; β2 represents the causal effect of HLA-DR⁺ mDC on Multiple myeloma (MM); β3 represents the total causal effect of EBNA-1antibody on MM. Indirect effect = β1Xβ2. Proportion mediated was the indirect effect divided by the total effect (Proportion mediated = β1Xβ2/β3).

We performed dual-sample MR analysis with 731 immune cell phenotypes with MM and identified 27 potentially positive mediating immune cell phenotypes (Supplementary Table 8). We further explored the mediating role of potentially positive mediators in the causal relationship between EBNA-1 antibody and MM using two-step MR. Two-step MR revealed that HLA-DR on myeloid dendritic cells (HLA-DR⁺ mDC) mediated the causal relationship between EBNA-1 antibody and MM (Supplementary Table 9). Specifically, EBNA-1 antibody increased the risk of MM by decreasing levels of HLA-DR⁺ mDC (Fig. 4).

## Discussion

EBV, also known as human herpesvirus 4, is a DNA virus within the herpesvirus family and one of the most widely distributed viruses globally^17^. EBV infection is generally asymptomatic or causes mild symptoms, though in some individuals it can lead to infectious mononucleosis, characterized by fever, sore throat, lymphadenopathy, and fatigue. Although previously considered largely harmless, emerging data clearly underscore the pathological role of lifelong EBV infection in driving autoimmunity and malignancy in a small yet significant subset of the population^37^. Since its discovery in 1964 as the first human oncogenic virus^38^, EBV infection has been linked to an increased risk of several cancers, accounting for approximately 1.5% of all human cancer cases worldwide^39^. Its associations with nasopharyngeal carcinoma^10^, gastric cancer^40^, and Hodgkin’s lymphoma^11^ are particularly well-established. The discovery of EBV laid the foundation for subsequent research on human oncogenic viruses, providing crucial insights into cancer prevention, diagnosis, and treatment. Observational studies have suggested an association between EBV infection and the incidence of MM, yet the causal relationship remains uncertain^12–16^.

The results of our two-sample MR analysis suggest that elevated EBNA-1 antibody levels are associated with an increased risk of MM, an observation validated across two independent datasets (FinnGen R11 and R10). While the initial association did not remain statistically significant after FDR correction, further MVMR analysis adjusting for potential confounders confirmed the causal effect of EBNA-1 antibodies on MM. These findings underline the robustness of the association and its independence from common confounding factors. Reverse MR analysis revealed no evidence supporting reverse causality, ruling out the possibility that MM influences EBV serostatus. This reinforces the hypothesis that chronic EBV infection, reflected in elevated EBNA-1 antibody levels, precedes and potentially contributes to MM development. Furthermore, two-step MR analysis identified HLA-DR⁺ mDC as a key mediator in this pathway. The downregulation of HLA-DR on mDCs by EBNA-1 antibodies supports the notion that chronic EBV infection may compromise antigen presentation and immune surveillance, thereby facilitating the development of MM. This mechanistic insight aligns with growing evidence that dysregulated immune responses play a pivotal role in MM pathogenesis.

EBNA-1 is a key nuclear protein encoded by the EBV that plays a critical role in EBV latent infection and pathogenic mechanisms. As one of the principal latency-associated proteins of EBV, EBNA-1 is essential for maintaining the viral genome and regulating host cell functions^37,41^. EBNA-1 interacts with host cells through multiple mechanisms, not only facilitating EBV latency but also playing a crucial role in the initiation and progression of various cancers^42^. EBNA-1 is expressed in latently infected B lymphocytes that are present throughout life in healthy carriers of the virus^43^. MM is a malignant tumour of the haematological system and is a malignant proliferative disease of plasma cells (a type of mature white blood cell differentiated from B cells). It has been found that EBNA-1 interacts with USP7, leading to ubiquitination and proteasomal degradation, which in turn destabilises p53, thereby promoting cell cycle progression and inhibiting apoptosis^44^. It has also been found that the EBV latent viral gene EBNA1 inhibits the cytotoxic response of NK cells by down-regulating the expression of NKG2D and c-Myc (a key protein in apoptosis) during the latent phase of B cells^45^. Moreover, the chronic inflammatory state associated with persistent EBV infection could contribute to the clonal expansion of plasma cells, ultimately leading to MM. This hypothesis is consistent with the “two-hit” model of MM pathogenesis, where a combination of genetic and environmental factors, such as chronic infections, drive disease progression^46^. Furthermore, the known effects of EBNA-1 on immune function support the biological plausibility of our findings. Several studies have found that the Gly-Ala repeat sequence (GAr) within EBNA1 inhibits the ability to present MHC class I-restricted antigens, a very important mechanism in the immune system that helps CD8 + T cells to recognise and clear abnormal (e.g. tumour) or infected cells^47–49^.HLA-DR⁺ mDC are myeloid-derived dendritic cells that express MHC II molecules and are primarily responsible for presenting exogenous antigens to CD4⁺ T cells to initiate an adaptive immune response. Additionally, our identification of HLA-DR⁺ mDCs as a mediator aligns with prior research showing the critical role of dendritic cells in maintaining immune surveillance against malignancies^50^. The downregulation of HLA-DR expression on dendritic cells could impair antigen presentation, a known mechanism through which EBV contributes to immune evasion and potentially oncogenesis^51^. Although we have not yet found studies specifically addressing the downregulation of HLA-DR⁺ mDCs by EBNA-1, it is known that EBV primarily infects B cells, including plasma cells responsible for antibody production. We speculate that the EBNA-1 antibodies produced by EBV-infected plasma cells may be recognized and bound by mDCs (a scenario that may occur in the context of immune dysregulation), leading to the suppression of HLA-DR expression. This downregulation may reduce the ability of mDCs to present external antigens, potentially limiting the activation of CD4 + T cells and thereby affecting the overall effectiveness of the immune response. This mechanism may represent one of the strategies by which EBV maintains latent infection in the host, allowing the virus to persist without eliciting a significant immune response. Ultimately, the EBV-infected B cells (such as the plasma cells producing EBNA-1 antibodies) may undergo abnormal proliferation, leading to the development of multiple myeloma. In summary, EBNA-1 disrupts immune system functions and regulates cell proliferation and apoptosis through multiple mechanisms, ultimately promoting the development and progression of multiple myeloma.

The strength of our study lies in the comprehensive and systematic evaluation of EBV infection and MM risk, as well as the exploration of the potential mediating role of Immune cell phenotype and the possible mechanisms underlying these relationships. However, this study also has several limitations. First, the study population was limited to individuals of European ancestry, which may restrict the generalizability of the findings to other populations. Second, although we employed various sensitivity tests to validate our results, we could not test the independence assumption or exclusion restriction in MR analysis, so the possibility of pleiotropic effects cannot be entirely ruled out. Third, the GWAS data used were summary-level data without individual-specific information, which prevented us from conducting subgroup analyses. Fourth, our analysis focused solely on cancer risk rather than disease progression, which limits our ability to provide insights into the potential utility of targeted biomarkers in cancer treatment. Fifth, our study only analyzed the relationship between potential antibodies to five EBV infection-associated proteins and MM; there are many other potential protein (EBNA 2, 3, and LMP)-derived antibodies to EBV infection, and a comprehensive assessment of them would likely reveal a more all-encompassing picture of the relationship between EBV infection and MM risk. Given these limitations, further experimental and clinical studies are needed to validate these findings and determine their potential value in clinical practice.

## Conclusions

The findings of this study suggest that EBNA-1 antibodies may increase the risk of MM by downregulating HLA-DR⁺ mDC. This indicates that chronic EBV infection may contribute to an elevated risk of MM. We hope these results provide new insights for future research on the prevention and treatment of MM.

## Electronic supplementary material

Below is the link to the electronic supplementary material.

Supplementary Material 1

Supplementary Material 2

Supplementary Material 3

## Data Availability

All data generated or analysed during this study are included in this published article [and its supplementary information files].

## Abbreviations

AEB-IgG: Anti-Epstein-Barr virus IgG seropositivity
cML: Constrained maximum likelihood
EA-D Ab: Epstein-Barr virus EA-D antibody
EBNA-1Ab: Epstein-Barr virus EBNA-1 antibody
EBV: Epstein-Barr virus
FDR: False discovery rate
FE-IVW: Fixed-effects Inverse variance weighted
GWAS: Genome-wide association study
IVs: Instrumental variables
IVW: Inverse variance weighted
LD: Linkage disequilibrium
MM: multiple myeloma
MR: Mendelian randomization
MVMR: Multivariate Mendelian randomization
RAPS: Robust adjusted profile score
RE-IVW: Random-effects Inverse variance weighted
SNP: Single nucleotide polymorphism
VCA-p18 Ab: Epstein-Barr virus VCA p18 antibody levels
WM: Weighted median
ZEBRA: Epstein-Barr virus ZEBRA antibody levels
