# Supplementary material for: Genetic evidence identifies a causal relationship between EBV infection and multiple myeloma risk
Source: Sci Rep. 2025 Feb 21;15:6357. doi: 10.1038/s41598-025-90479-1 (PMC11845450; doi:10.1038/s41598-025-90479-1)
Supplement: Supplementary file 3 — Supplementary Material 3 [file 41598_2025_90479_MOESM3_ESM.pdf]

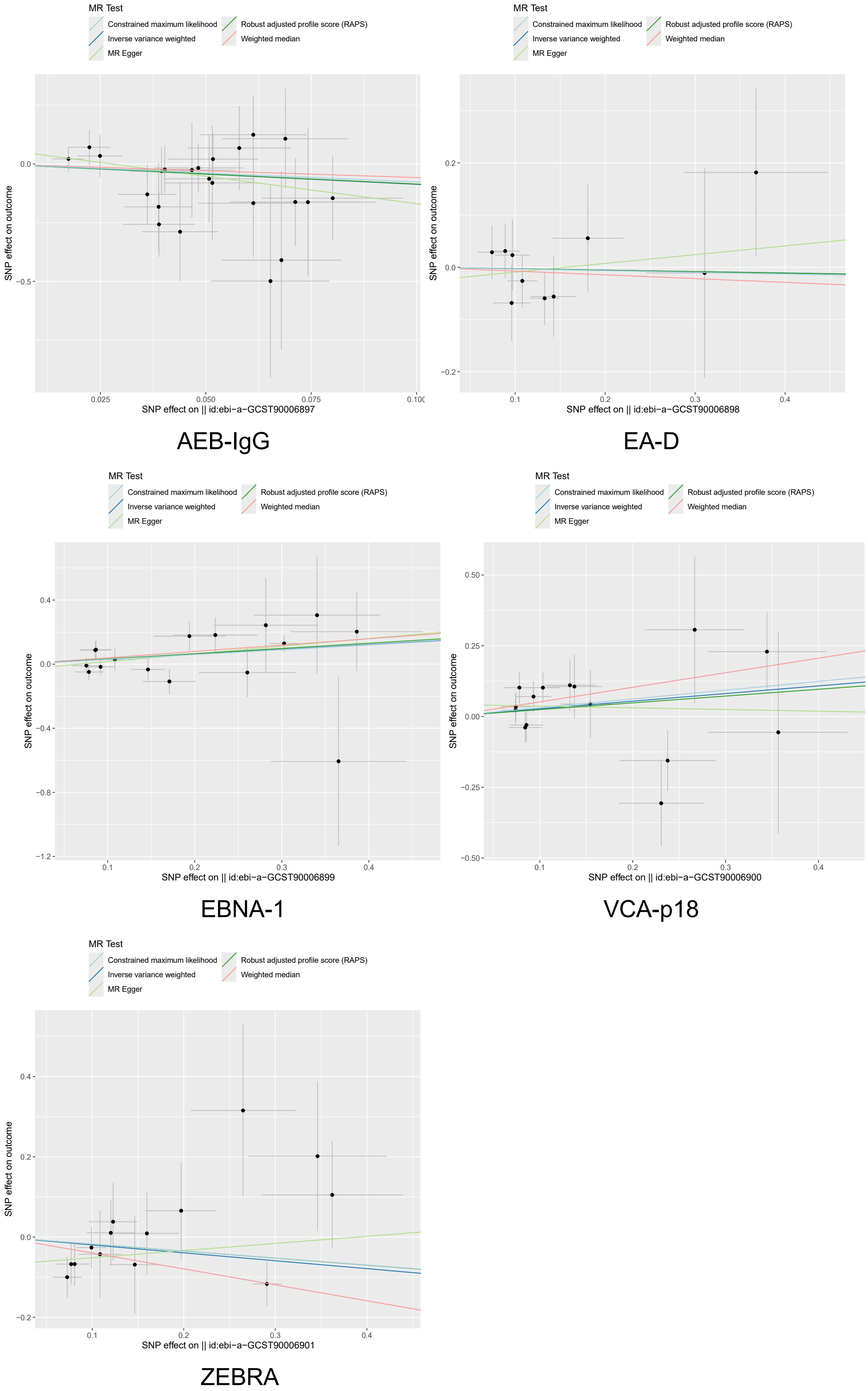

(A). Scatter plot of EBV-associated antibodies on MM(R11).

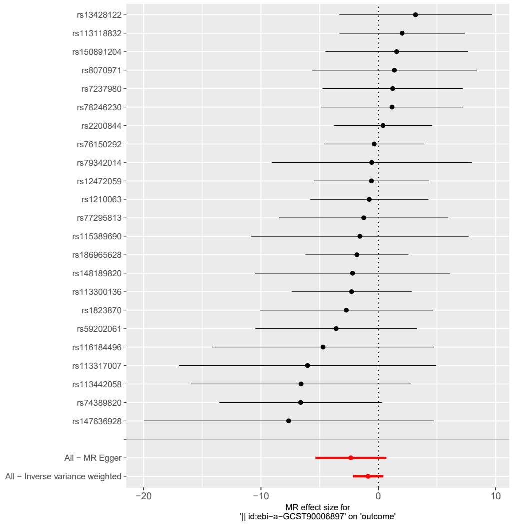

AEB-IgG

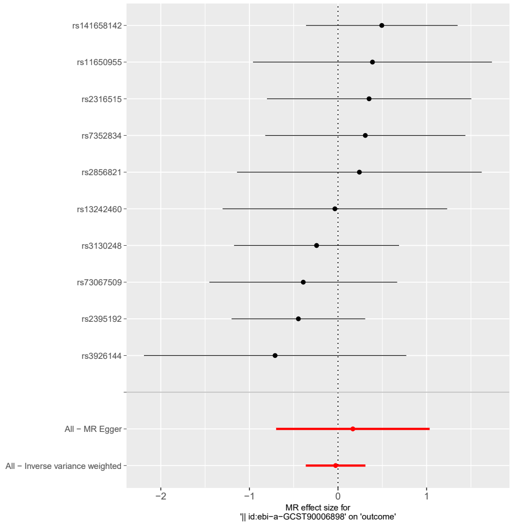

EA-D

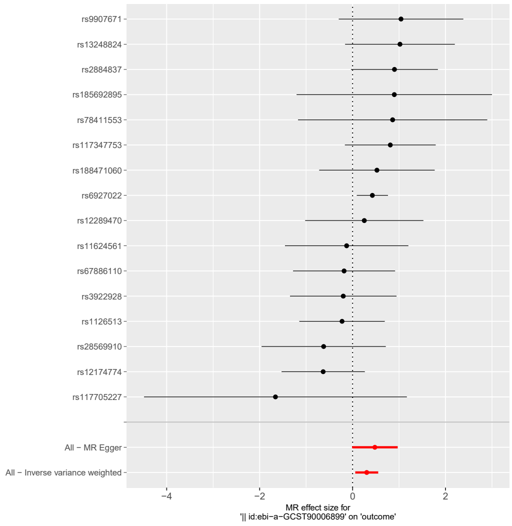

EBNA-1

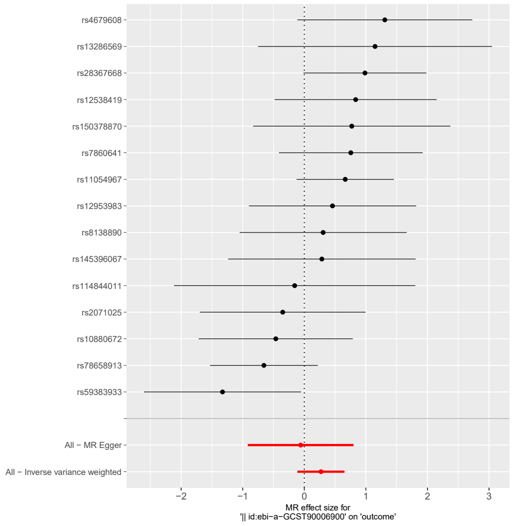

VCA-p18

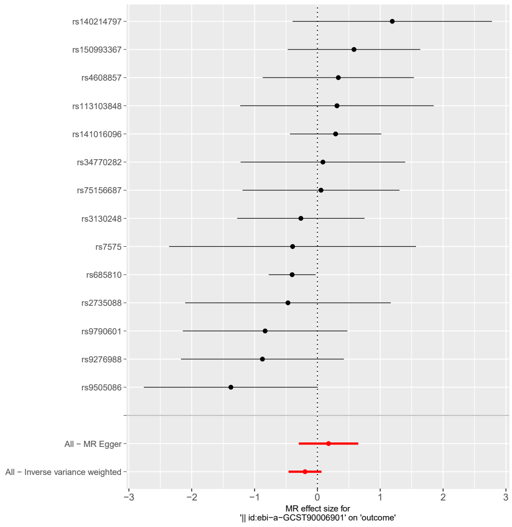

ZEBRA

(B). Forest map of EBV-associated antibodies on MM(R11).

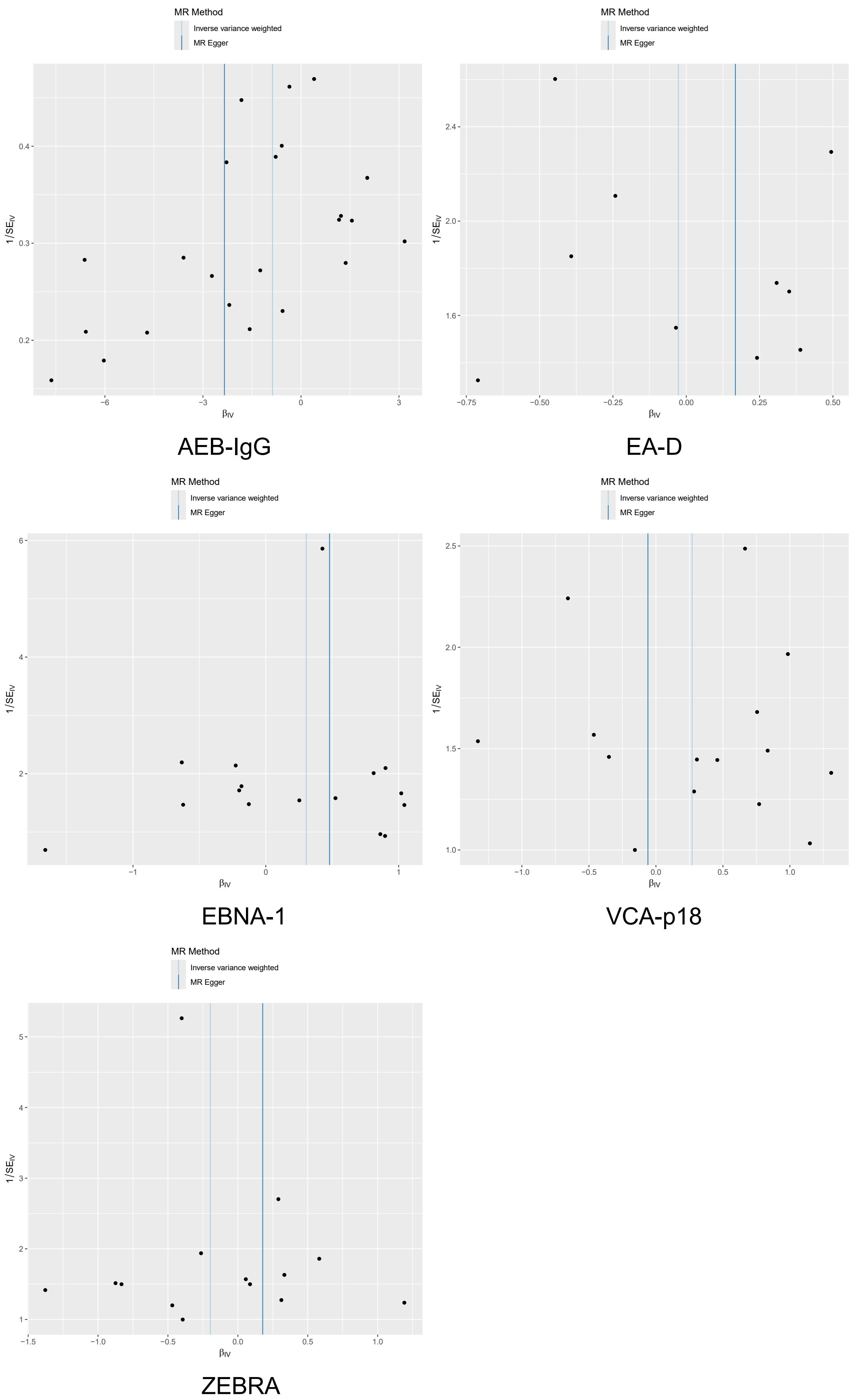

(C). Funnel plot of EBV-associated antibodies on MM(R11).

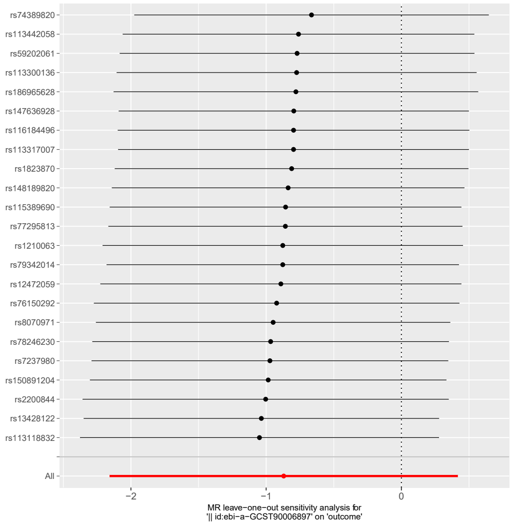

AEB-IgG

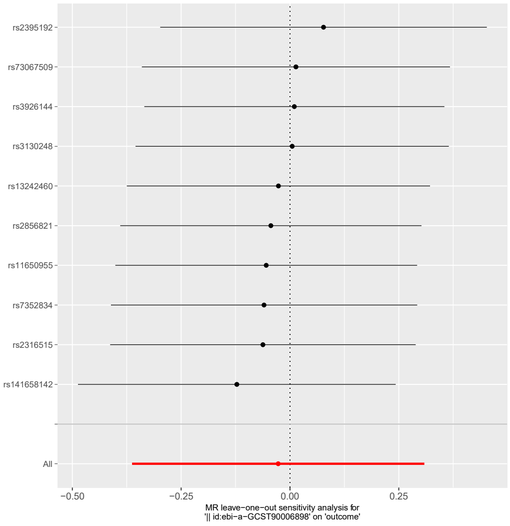

EA-D

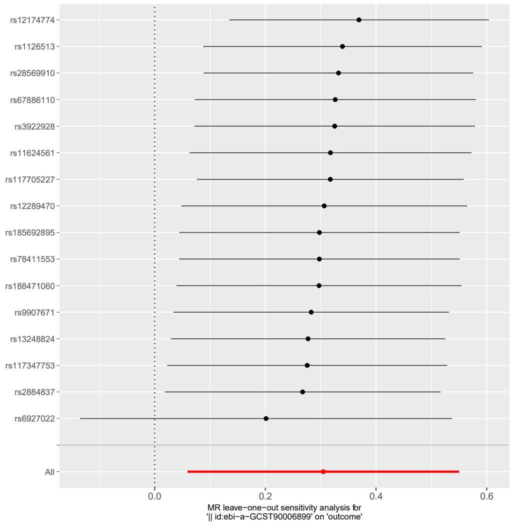

EBNA-1

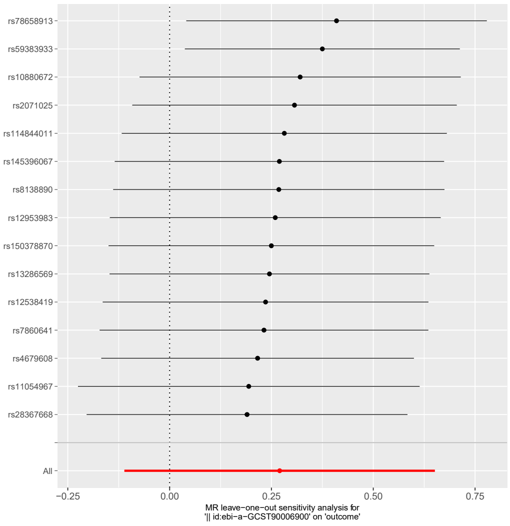

VCA-p18

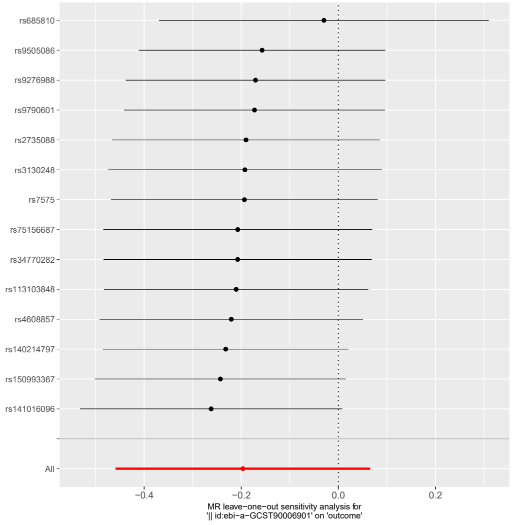

ZEBRA

(D). Leave-one-out of EBV-associated antibodies on MM(R11).

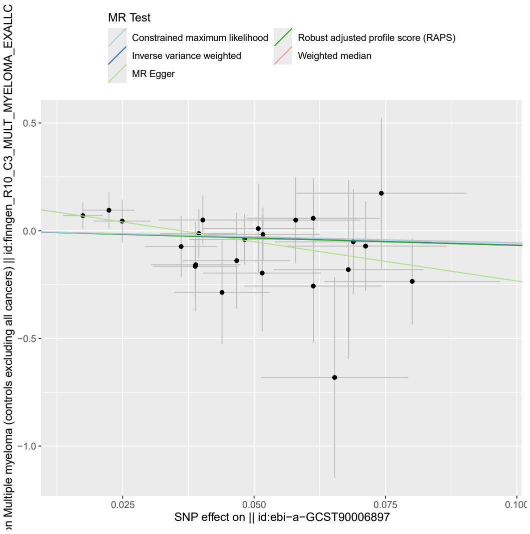

AEB-IgG

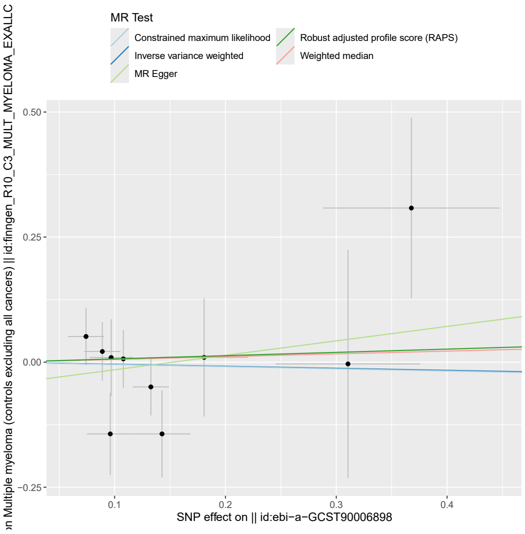

EA-D

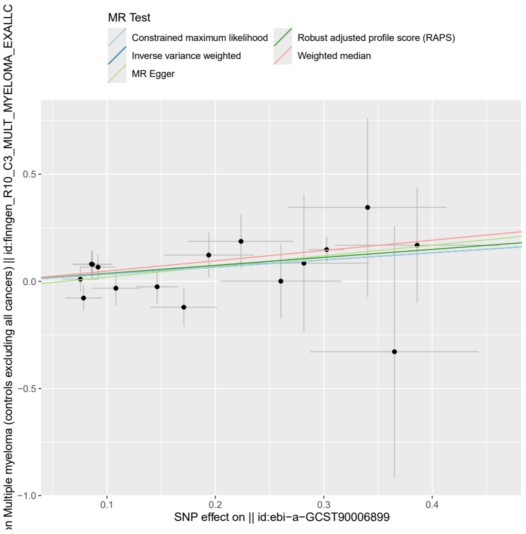

EBNA-1

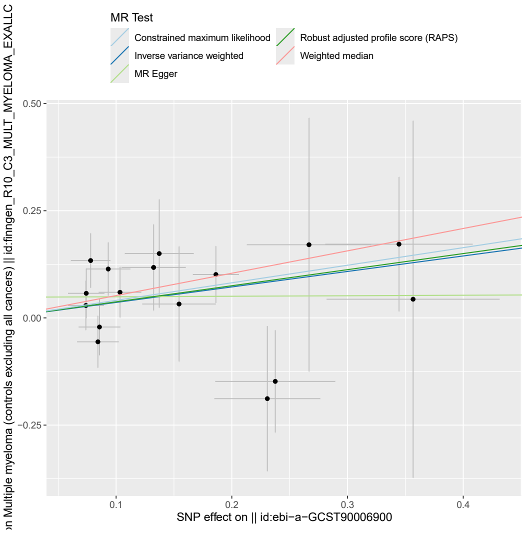

VCA-p18

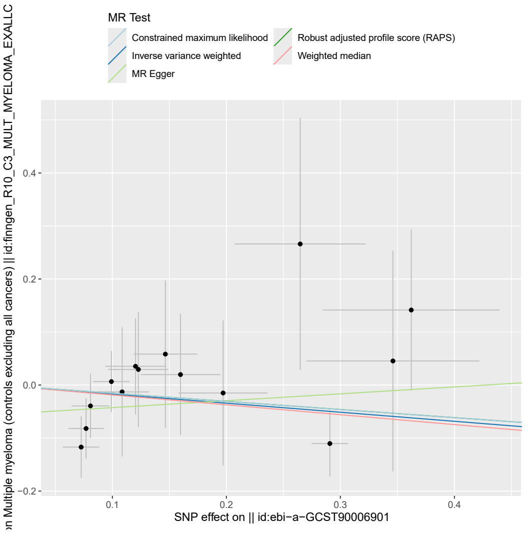

ZEBRA

(E). Scatter plot of EBV-associated antibodies on MM(R10).

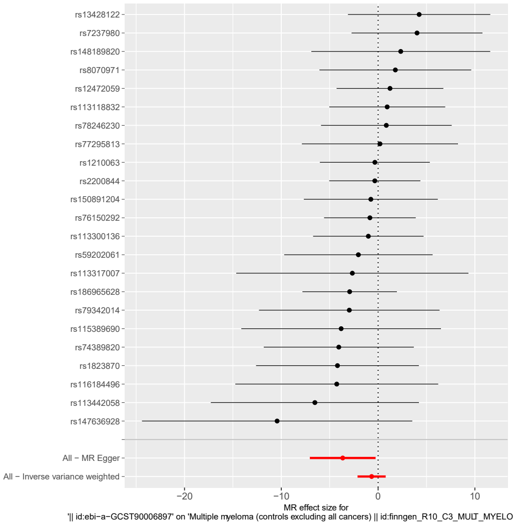

AEB-IgG

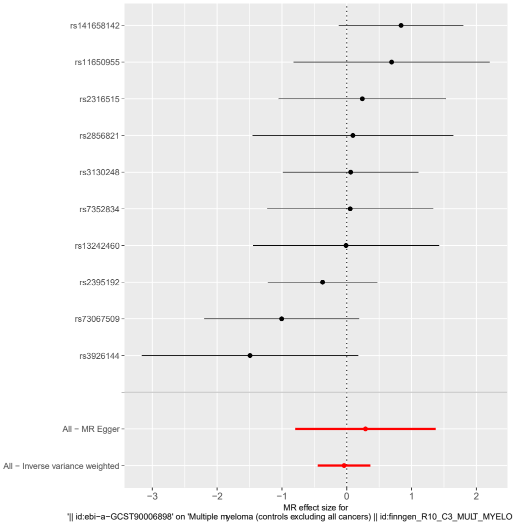

EA-D

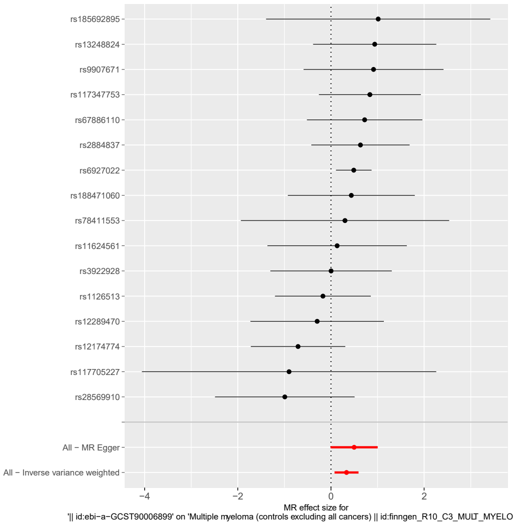

EBNA-1

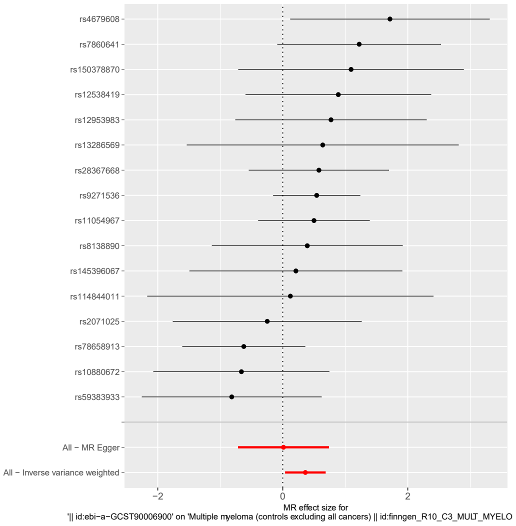

VCA-p18

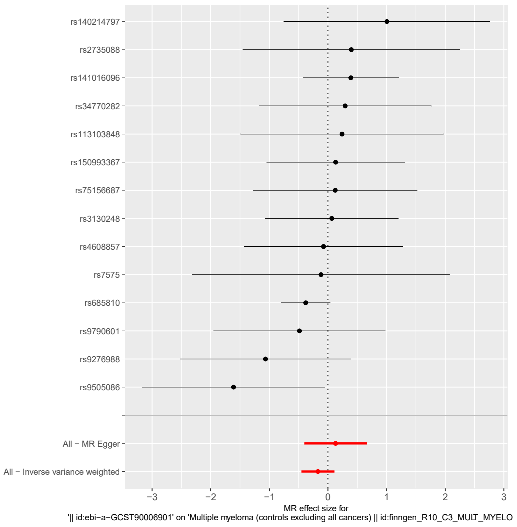

ZEBRA

(F). Forest map of EBV-associated antibodies on MM(R10).

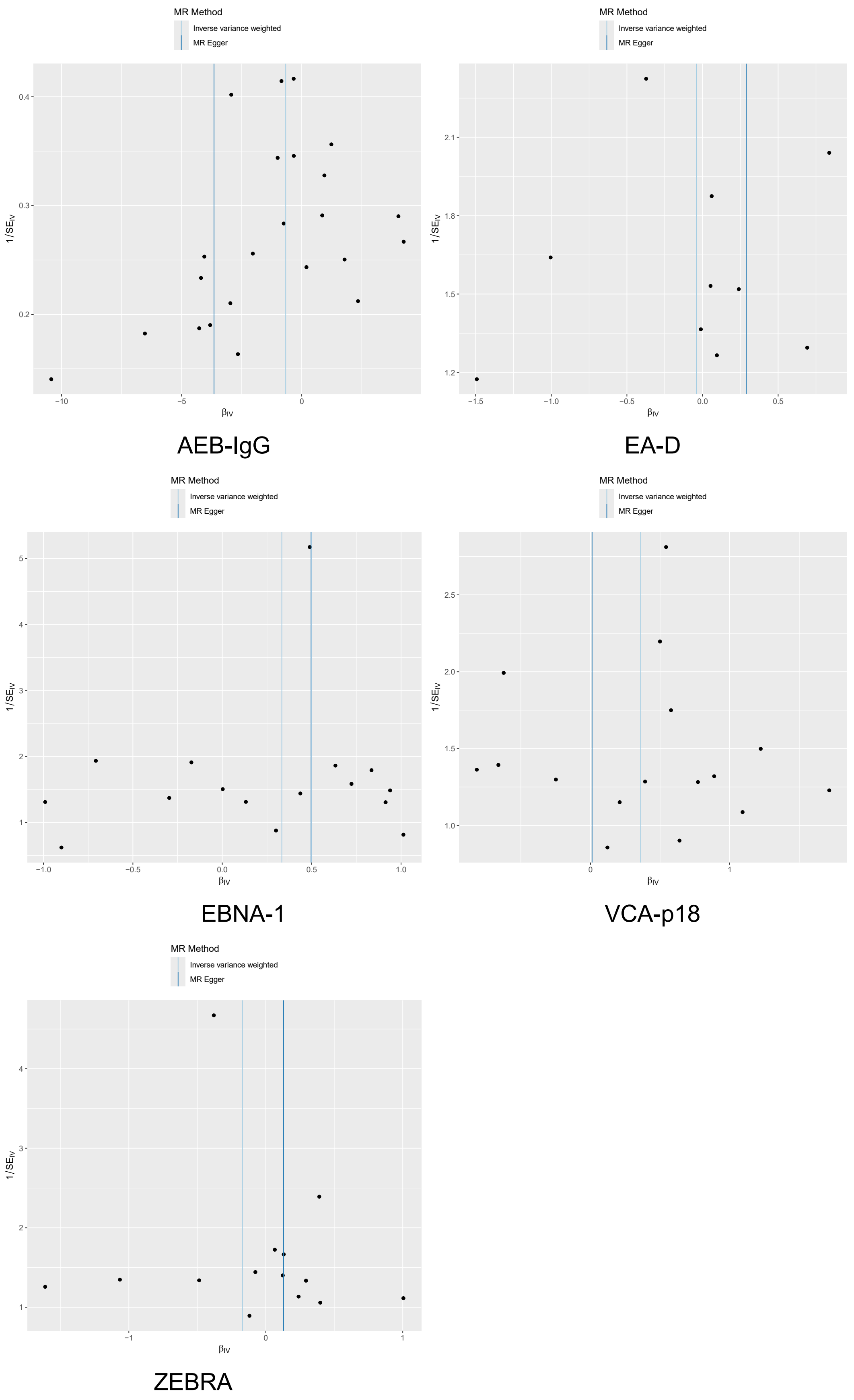

(G). Funnel plot of EBV-associated antibodies on MM(R10).

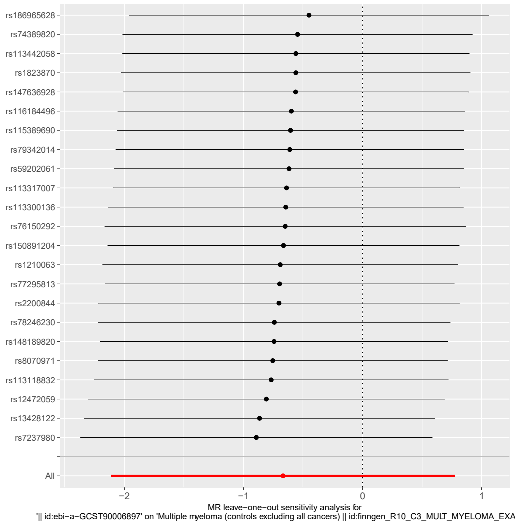

AEB-IgG

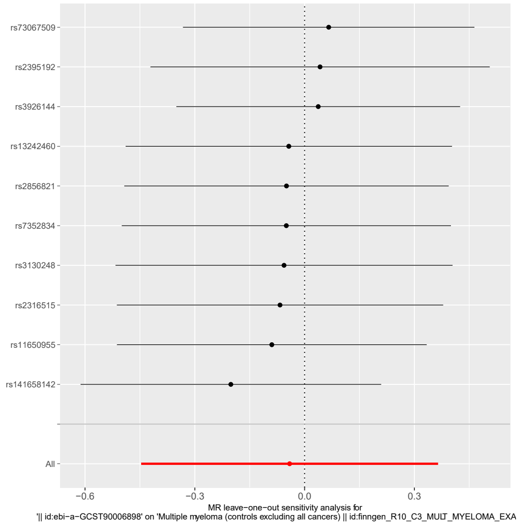

EA-D

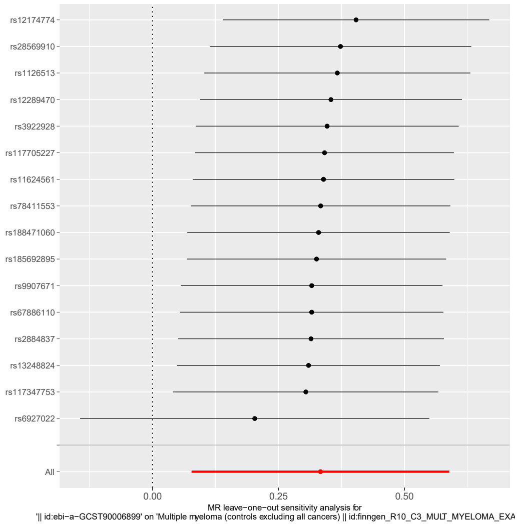

EBNA-1

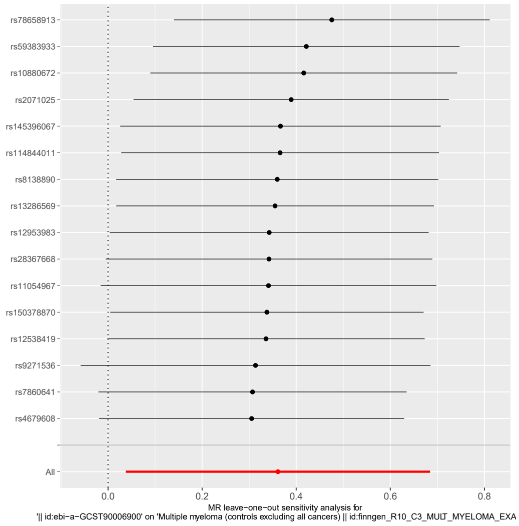

VCA-p18

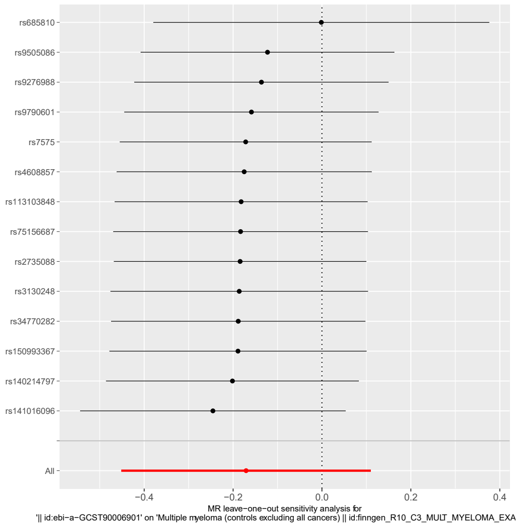

ZEBRA

(H). Leave-one-out of EBV-associated antibodies on MM(R10).

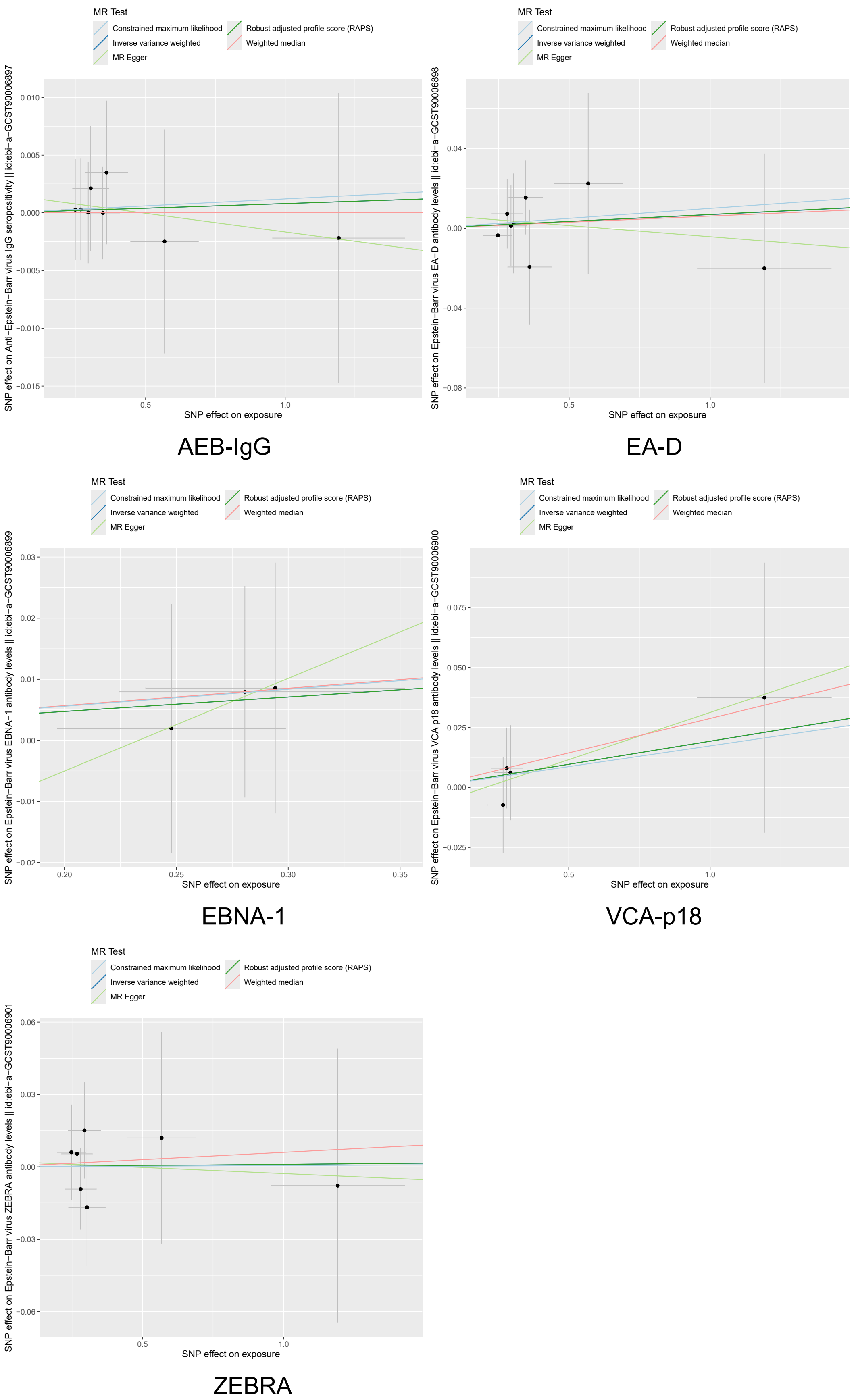

(I). Scatter plot of MM(R11) on EBV-associated antibodies.

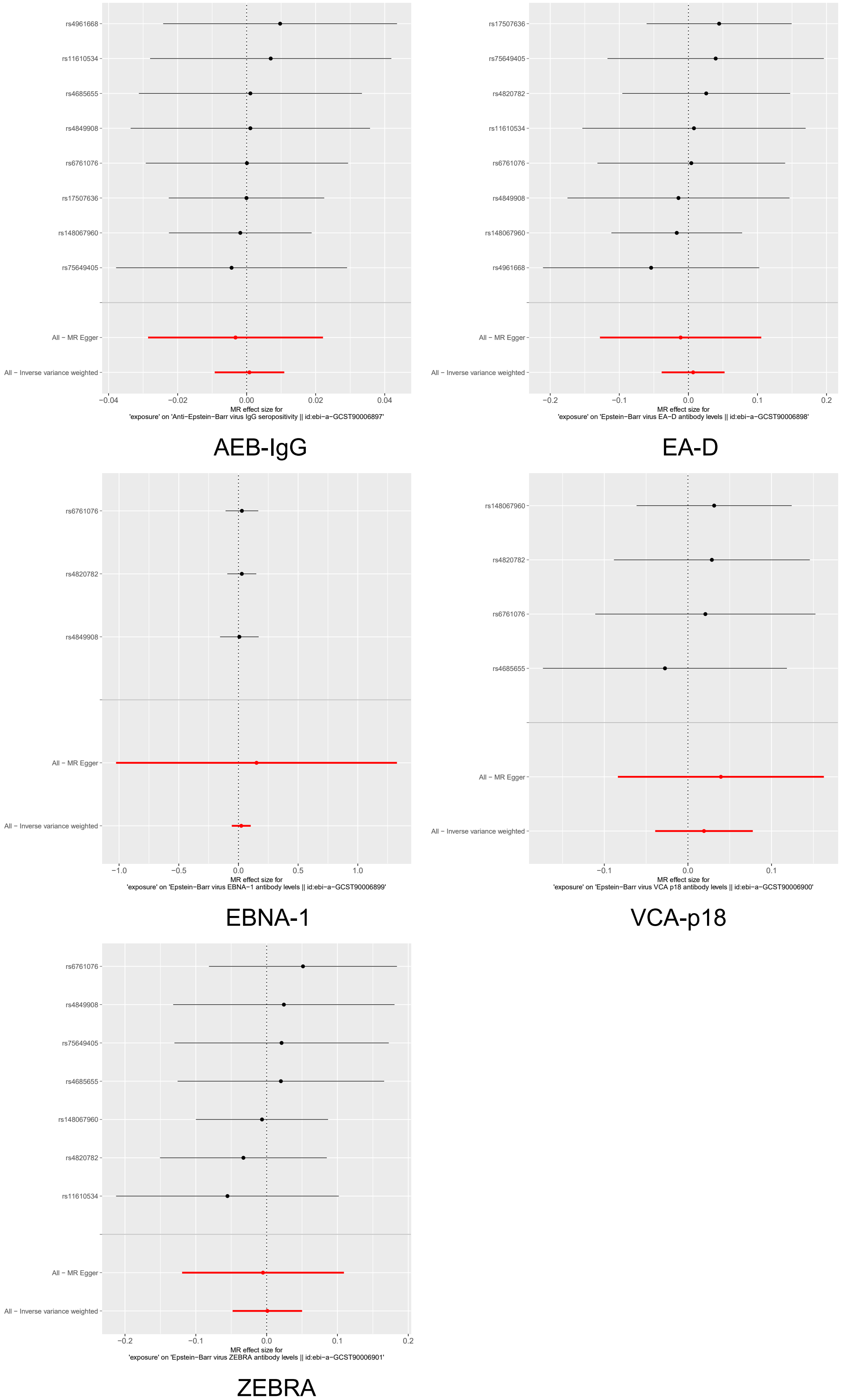

(J). Forest map of MM(R11) on EBV-associated antibodies.

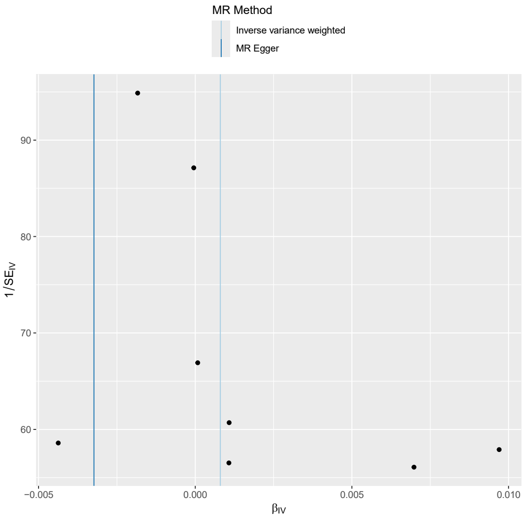

AEB-IgG

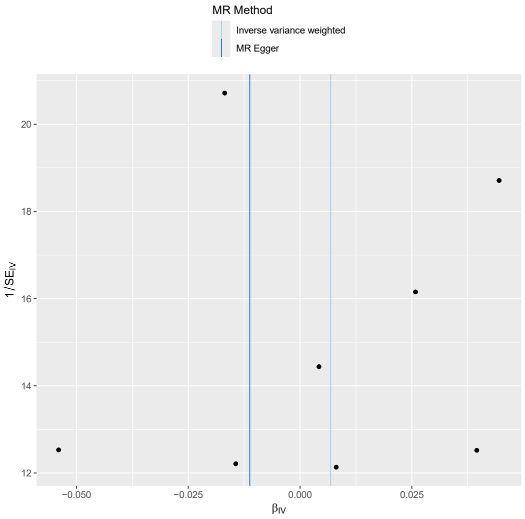

EA-D

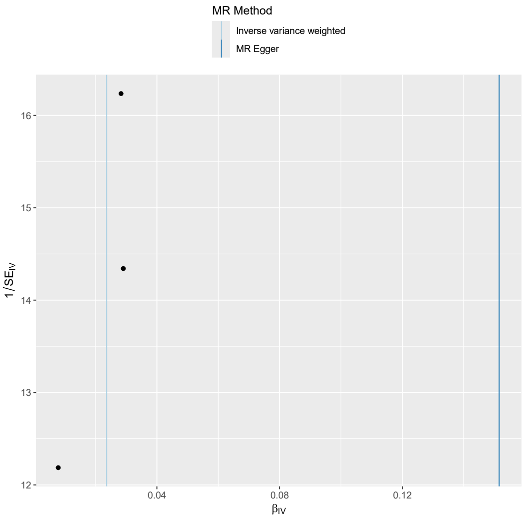

EBNA-1

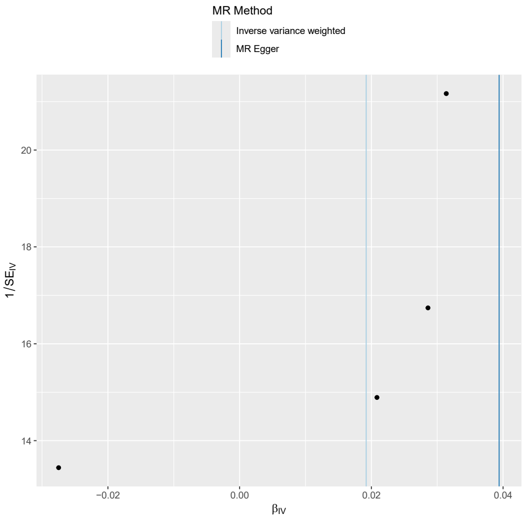

VCA-p18

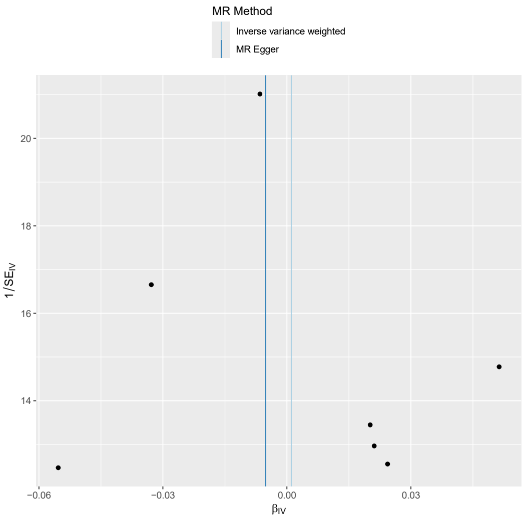

ZEBRA

(K). Funnel plot of MM(R11) on EBV-associated antibodies.

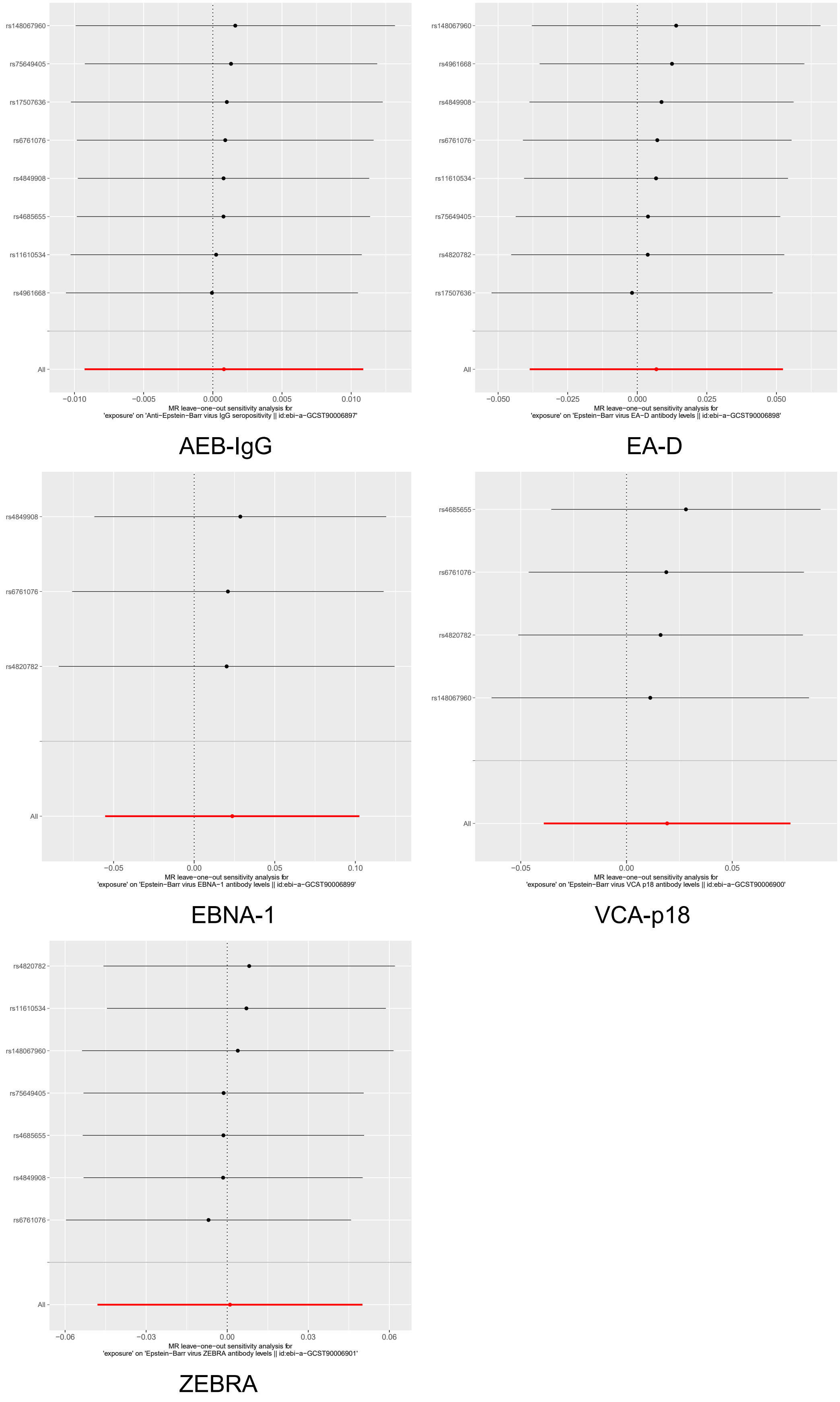

(L). Leave-one-out of MM(R11) on EBV-associated antibodies.

Supplementary Figure 1. Two-sample Mendelian randomization analysis scatter plot, forest plot, funnel plot and leave-one-out method. (A). Scatter plot of EBV-associated antibodies on MM(R11); (B). Forest map of EBV-associated antibodies on MM(R11); (C). Funnel plot of EBV-associated antibodies on MM(R11); (D). Leave-one-out of EBV-associated antibodies on MM(R11); (E). Scatter plot of EBV-associated antibodies on MM(R10); (F). Forest map of EBV-associated antibodies on MM(R10); (G). Funnel plot of EBV-associated antibodies on MM(R10); (H). Leave-one-out of EBV-associated antibodies on MM(R10); (I). Scatter plot of MM(R11) on EBV-associated antibodies; (J). Forest map of MM(R11) on EBV-associated antibodies; (K). Funnel plot of MM(R11) on EBV-associated antibodies; (L). Leave-one-out of MM(R11) on EBV-associated antibodies.
